# Incidence, Therapy, and Bleeding Risk—Cancer- Associated Thrombosis in Patients with Glioblastoma

**DOI:** 10.3390/cancers12061354

**Published:** 2020-05-26

**Authors:** Viktoria Muster, Thomas Gary

**Affiliations:** Division of Vascular Medicine, Department of Internal Medicine, Medical University of Graz, 8036 Graz, Austria; thomas.gary@medunigraz.at

**Keywords:** venous thromboembolism, glioblastoma, cancer-associated thrombosis, anticoagulation, risk factors, treatment

## Abstract

Cancer is an independent risk factor for the development of venous thromboembolism (VTE). Glioblastomas are amongst cancer types with the most thrombogenic potential and patients are at a particularly high risk of VTE with an incidence up to 20–30% per year. Currently, major efforts are underway to gain novel insights into risk factors and pathomechanisms to provide a better understanding of development of VTE in patients with primary brain tumors. Treatment of VTE requires therapeutic anticoagulation, which accordingly to recently-published guidelines should be performed using low molecular weight heparin or, in case of low bleeding risk, using a direct oral anticoagulant. However, this can be very challenging due to an increased risk of intracranial hemorrhage in this patient group. Furthermore, limited data are available on the subgroup of patients with primary brain tumors.

## 1. Introduction

Cancer is an independent risk factor for the development of venous thromboembolism (VTE), including deep vein thrombosis and pulmonary embolism, and leads to a four-fold increase in the risk of VTE in this patient group [1]. Depending on the cancer site, primary brain tumors, especially glioblastoma, belong to the group of cancer types with the most thrombogenic potential. As the most malignant human brain tumor, glioblastoma morphologically show an infiltrative growth, extreme neoangiogenesis, intratumoral necrosis, and intravascular thrombosis as well as endothelial cell proliferation [2]. Glioblastoma patients are at a particularly high risk of VTE with an incidence up to 20–30% per year [3,4]. An association with the 1-year relative mortality can be clearly seen between the biological aggressiveness of the cancer and its thrombogenic potential [3,4,5,6,7]. In this patient group, VTE is a leading cause of death and patients with VTE have a higher mortality than those without VTE [8,9]. 

## 2. Risk Factors and Pathomechanisms

**Figure 1:**
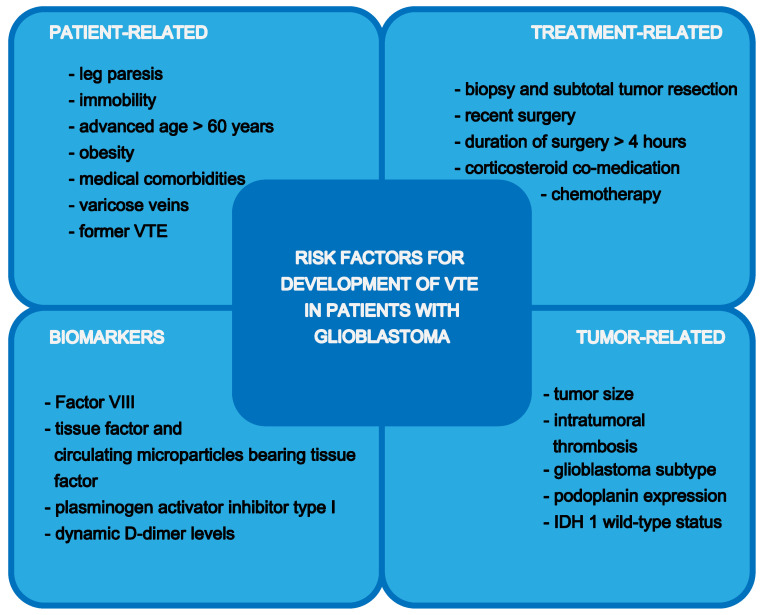
Risk factors for development of venous thromboembolism (VTE) in patients with glioblastoma.

Risk factors for the development of VTE in patients with glioblastoma can be grouped in patient-, treatment-, and tumor-related risk factors, which also supports the determination of the individual risk for a patient to develop VTE (Figure 1).

Postoperatively, the incidence of VTE is highly variable, mainly due to the use of different prophylaxis regimens. However, the risk of VTE is not only present in the postoperative period but remains high throughout the course of the malignancy, with most VTEs occurring three to six months after cancer diagnosis [10,11].

Due to a high rate of diagnosis of VTE in the preoperative as well as in the perioperative setting, the importance of frequent monitoring and taking preventive measures needs to be highlighted [12].

Nevertheless, not all mechanisms of potential risk factors for the development of VTE in glioblastoma patients are yet fully understood and continuous research in this field is of enormous value with a view to gaining new insights.

### 2.1. Patient-Related Risk Factors

Possible risk factors for the development of VTE in cancer patients are the presence of leg paresis, advanced age > 60 years, obesity and medical comorbidities such as renal and hepatic disease, infection, congestive heart failure, arterial thromboembolism, and anemia [10,11,13]. The existence of varicose veins or previous VTE are also risk factors for the development of VTE in these patients [14,15].

In the RIETE (Computerized Registry of Patients with Venous Thromboembolism) registry it was found that patients with glioblastoma were immobile more often in comparison to patients with other cancer types (32% vs. 19%) and had an earlier occurrence of VTE after the diagnosis of cancer [16].

### 2.2. Tumor-Related Risk Factors

Compared to lower-grade glioma, the histologic diagnosis of glioblastoma multiforme as well as a large tumor size is associated with an increased risk of VTE in patients with primary brain tumors [10]. There is evidence that morphological processes lead to tumor-induced hypercoagulability in patients with glioblastoma. Rodas et al. for example point out that in patients with VTE and malignant glioma, a higher rate of intratumoral thrombosis can be detected [17].

In the Vienna Cancer and Thrombosis Study (CATS) the rate of VTE was nearly twice as high in patients with high grade tumors compared to those with low grade tumors after adjusting for the cofactors of distant metastases, sex, and age (hazard ratio (HR) (95% CI), 2.0 (1.1–3.5); *p* = 0.015) [18]. The CATS also showed a high tumor podoplanin expression, which correlated with a laboratory measured hypercoagulability state, intratumoral thrombosis, and increased VTE risk [19]. Results of future in vivo studies may confirm this promising hypothesis.

The study conducted by Unruh et al. found an association between a lower risk of VTE and the isocitrate dehydrogenase 1 (IDH1) mutation in patients with glioblastoma, which was also confirmed in an analysis of the Vienna CATS trial [20,21]. Additionally, it was found that excessive podoplanin expression only occurred in IDH1 wild-type tumors while in IDH1 mutant tumors podoplanin overexpression could not be found. This finding enabled a risk stratification for VTE in these patients which resulted in a risk of 0% after 6 months for patients with an IDH1 mutant tumor and no podoplanin expression versus 18.2% in patients with IDH1 wild-type tumor and high podoplanin expression [21].

### 2.3. Treatment-Related Risk Factors

For patients with high-grade glioma, it was observed that postoperatively after biopsy and subtotal tumor resection, the frequency of VTE was higher compared to patients with total tumor resection [7]. This was also found in a prospective multicenter study published by Streiff et al. where patients with initial tumor biopsy had a 3-fold higher risk of developing VTE [2]. Recent surgery was also found as a treatment-related risk factor in patients with glioblastoma in the RIETE registry compared to those with other cancer types (36% vs. 14%) [16].

Furthermore, the use of chemotherapy and duration of surgery > 4 h can be named as treatment-related risk factors [10].

A very high percentage of patients with glioblastoma were receiving corticosteroids at the time of the VTE diagnosis (70% vs. 13%) but were less likely to be using antiplatelet drugs which can be explained by the significantly younger age and less chronic diseases in patients with glioblastomas compared to other cancer types [16].

### 2.4. Biomarkers

The finding of a suitable biomarker to assess the risk of occurrence of VTE in patients with brain tumors is of utmost clinical interest. Promising findings are being published by a number of different study groups which may lead to clinical implications in the near future.

Streiff et al. found in their prospective multicenter study, that patients with high grade glioma and elevated factor VIII levels, had a 2.1-fold increase of risk for the development of VTE. However, issues focused on in previous studies such as ABO blood group, D-dimer levels, and thrombin generation were not found to increase the risk of VTE in this study [2]. 

On the other hand, Hoke et al. as well as Navone et al. found a reduction in overall survival and disease-free survival and an increased risk of thromboembolic events associated with high D-dimer levels in glioblastoma patients [22,23].

A more innovative approach to biomarker measurement for VTE risk prediction might be a longitudinal measurement, for example of D-dimer levels, as presented by Posch et al. In their study it was hypothesized that a single measurement of a biomarker may only represent a “snapshot” of cancer-associated hypercoagulability at a single time point, not taking into account that patient-related, tumor-related, and therapy-related risk factors can change over the course of the disease and thus that personal high-risk time to develop VTE can also change. By a longitudinal measurement of a biomarker and the use of joint models, a highly personalized dynamic prediction of VTE risk may be obtained. The authors found that D-dimer levels may be elevated in a specific patient but remain stable over time if no VTE event occurred. For patients who developed VTE, a strong increase in D-dimer levels was found during follow-up. In this study several tumor entities were analyzed including 50 primary brain tumor patients. Whether the same strength of association between longitudinal measured D-dimer levels and risk for VTE can also be shown in individual tumor entities or if other biomarkers have a tumor-specific relevance may be explored by future studies [24].

As possible pathomechanism of VTE in patients with brain tumors, tissue factor and plasminogen activator inhibitor type I, which are among the procoagulant and antifibrinolytic substances, as well as circulating microparticles bearing tissue factor can be found more frequently in high grade glioma than in other brain tumors, and are therefore discussed by several authors [25,26,27,28,29].

Recently it was found that, for patients with glioblastoma, compared to patients with meningioma, a shorter prothrombin time and activated partial thromboplastin time is associated with a reduced overall survival. Most probably this finding is due to their state of hypercoagulability. Moreover, higher plasma levels of von Willebrand factor result in a 3-fold increase of risk of death and are associated with a reduced survival in glioblastoma patients compared to patients with meningioma [23].

## 3. Bleeding Risk and Anticoagulation

Especially in patients with primary brain tumors, intracranial hemorrhage (ICH) is also observed without the administration of anticoagulation [30,31]. There are also several studies that point out that the tumor, through angiogenesis mediators such as vascular endothelial growth factor (VEGF) and matrix metalloproteinases (MMPs), implicates ICH itself. Further preclinical and clinical data is needed to fully understand the roles of these mediators in the pathogenesis of cancer-associated ICH [32].

Furthermore, there is only limited data available for this patient group regarding bleeding risk in case of anticoagulation. This often leaves the clinician with a high degree of uncertainty about the best treatment option for each patient.

Lee et al. found in their CLOT study a better efficacy and safety of low-molecular-weight heparin (LMWH) compared with vitamin-k antagonists for the treatment of cancer-associated venous thromboembolism, which, for nearly two decades, lead to the recommendation to choose anticoagulation with LMWH over a vitamin-k antagonist in the absence of contraindications [33,34]. However, only 27 patients with brain tumors were included in this study with no outcome data for this subgroup reported.

Due to the lack of evidence, further investigations were initiated and retrospective studies were conducted analyzing the relationship between anticoagulation for VTE and the risk of ICH in patients with glioblastoma. These studies reported an ICH rate range between 0% and 12% [4,35,36,37]. 

In a more recent study, Khoury et al. showed, that 15.5% of patients receiving anticoagulation developed ICH compared to 2.6% of patients who were not receiving anticoagulation. Yet, in this study, neurosurgical intervention was not needed for the majority of patients with ICH. Compared to previously-mentioned studies, Khoury et al. exclusively involved patients with WHO grade IV glioma. Furthermore, less sensitive cranial computer tomography (CT) scans were used in older studies compared to magnetic resonance imaging (MRI) scans that were performed in this study. These two facts may account for the higher rate of ICH-detection that was reported in this study. Furthermore, the authors found that patients receiving anticoagulation tended to have a longer post-VTE diagnosis survival and overall survival than patients with VTE who were not receiving anticoagulation. However, this may also imply that patients in a massively-reduced general condition were not able to receive anticoagulant treatment. These clinical factors, including patients’ age, led to a selection bias may have had an effect on patient survival. Furthermore, there was no association found between the incidence of ICH and the type of anticoagulation. Khoury et al. interestingly found in their study that the presence or size of enhancing tumor did not need be a contraindication for anticoagulation as no difference was found in patients with or without ICH. This is also consistent as no correlation between extent of surgical resection and ICH incidence was found [12].

In a meta-analysis, including the studies outlined above involving 539 anticoagulated patients (receiving warfarin, LMWH and warfarin, or LMWH alone), the authors found that the overall risk for ICH in patients with glioblastoma was more than 3-fold higher when receiving anticoagulation in comparison to those who were not receiving anticoagulation. Nevertheless, the overall incidence of fatal ICH in this meta-analysis was less than 1% [38].

However, the interpretation of these results is difficult because a major limitation of these studies is the lack of homogenous definition of ICH. ICH is defined from trace radiologic evidence of blood products to clinically-manifest hemorrhages with mass effect. Additionally, the diagnosis of ICH in patients with glioblastomas undergoing surgical procedures, which can lead to postoperative hemorrhage as a result of such procedures, leads to additional confounding. Mantia et al. therefore performed a retrospective cohort study with blinded radiology review and predefined ICH criteria. Two of the inclusion criteria in this study were WHO grade III or IV glioma (84% included were glioblastomas) and a therapeutic anticoagulation with enoxaparin for a diagnosis of VTE. One exclusion criterion was any bleed within 4 weeks after a neurosurgical procedure. An association between the administration of therapeutic enoxaparin and risk of developing a major ICH at 1 year was found to be greater than 3-fold in the enoxaparin group [39].

Publication of the data from the Hokusai VTE-cancer study marked the beginning of a new era of anticoagulation for cancer-associated VTE (Table 1). This was the first study to show a non-inferiority for the composite endpoint of bleeding and VTE recurrence in patients with cancer-associated VTE in a head-to-head analysis of LMWH and the direct oral anticoagulant (DOAC) edoxaban. While VTE recurrence was lower in the edoxaban group compared with dalteparin, the rate of major bleedings was higher in the edoxaban group. The majority of these bleedings were gastrointestinal bleedings which mostly occurred in patients with primary gastrointestinal tumors. Patients with primary brain tumor or brain metastases were also included in this study, but only accounted for a small number (*n* = 74) and no further analysis regarding tumor type nature or extend of bleeding was reported separately [40].

For the other two factor Xa direct oral anticoagulants, rivaroxaban and apixaban, similar studies were performed but included a much smaller number of patients.

The SELECT-D study, which compared rivaroxaban to dalteparin in patients with active cancer and cancer-associated VTE, found a lower recurrence rate of VTE but also a higher bleeding rate in the rivaroxaban group. However, only three patients with primary brain tumors were included in the study, two in the dalteparin group and one in the rivaroxaban group. No further subgroup analysis of this patient group is reported [41].

For apixaban, the Adam-VTE trial found that compared with dalteparin in patients with cancer-associated VTE, patients showed a low bleeding risk, similar to those without cancer, and there was also a lower recurrence rate in this group. In this study only a very small number (*n* = 8) patients with primary brain tumors were included [42].

Only recently, the Caravaggio trial showed that apixaban is comparable to dalteparin for the treatment of cancer-associated VTE. The risk of major bleeding was not increased in the apixaban group. However, patients with brain tumors were excluded from this study thus making it impossible to extrapolate the results reported for this patient group [43].

In conclusion, DOAC treatment seems to be associated with an elevated bleeding risk in cancer patients. However, due to the small sample size of brain tumor patients the question of whether DOACs also increase the risk of ICH has not been answered by studies conducted so far.

However, Carney et al. compared the rates of ICH in patients with brain tumors and cancer-associated VTE treated either with DOACs or LMWH in a retrospective analysis. Patients with grade III or IV glioma and two or more radiographic brain images to review were included in this analysis. Patients with ICH that occurred before the initiation of anticoagulation or within four weeks of neurosurgery were excluded. ICH was classified into trace, measurable, or major. A total of 67 patients with primary brain tumors were analyzed of which 20 patients received a DOAC and 47 patients received enoxaparin. In the DOAC patient group, five patients received rivaroxaban and 15 received apixaban. The cumulative incidence of ICH (including all three classifications) at 12 months was 0% in patients receiving DOAC treatment. Patients receiving enoxaparin had a cumulative incidence of ICH of 36.8% (95% confidence interval 22.3–51.3%) in this analysis, of which eight ICH were major ICH. The case-fatality for post-ICH 30-day mortality was 39% in the enoxaparin group and 0% in the DOAC group [44]. This study has several limitations: first the retrospective study design and second the selection of the patients. It was up to the treating clinicians to decide whether the patient was treated with a DOAC or with LMWH. Therefore, patients in a reduced general condition and therefore with high bleeding risk were more likely be selected for LMWH leading to a higher ICH risk. Nevertheless, Carney et al. point out an improved safety profile for the use of DOACs in high-risk patients with primary brain tumors. Therefore, DOAC treatment seems to be a reasonable option in case of cancer-associated VTE in this population [44].

## 4. Prophylaxis and Therapy According to Current Guidelines

### 4.1. Prophylaxis

Given the high risk of VTE in patients with glioblastoma throughout the course of the malignancy, several studies were conducted investigating whether a long-term pharmacological prophylaxis might be of benefit. Similar to studies considering the management of acute VTE in cancer patients with primary brain tumors, data for a prolonged prophylaxis is also scarce.

In a randomized controlled trial of patients with newly-diagnosed glioblastoma, 99 patients were allocated to receive 6 months of prophylactic dalteparin, starting within 4 weeks of surgery and 87 patients were allocated to receive placebo. The incidence of VTE was higher in patients in the placebo group (HR 0.51; 95% CI 0.19–1.4; *p* = 0.29), but the incidence of major bleeds was increased in the LMWH group (HR 4.2; 95% CI 0.48–36; *p* = 0.22). However, neither of the results reached statistical significance [45].

A meta-analysis of ten randomized controlled studies including 1263 patients with primary brain tumors undergoing craniotomy, analyzed the benefit–risk ratio of several prophylactic VTE measures. It was found that prophylactic VTE measures lead to a significantly lower risk of VTE while not causing a major increase in bleeding. Patients receiving unfractionated heparin alone had a stronger risk reduction in VTE than patients receiving placebo (RR = 0.27; 95% CI 0.1–0.73) and LMWH with mechanical prophylaxis together showed a lower VTE risk than mechanical prophylaxis alone (RR = 0.61; 95% CI 0.46–0.82) [46].

Concerning DOACs, their use for long-term prophylaxis of VTE in cancer patients was subject of two randomized controlled studies.

The CASSINI study randomized 841 patients with Khorana score >2, a risk score for VTE in cancer patients, but not well established for brain tumor patients [47], between rivaroxaban 10 mg once daily and placebo. The number of VTEs was lower in the rivaroxaban group compared to placebo (6% versus 8.8%) but did not reach statistical significance. Patients with brain tumors or brain metastases were excluded [48].

A similar population was randomized in the AVERT study, which included 574 patients, and compared apixaban 2.5 mg twice daily and placebo for long-term prophylaxis of VTE in cancer patients. In this study, a significant reduction of VTE in the apixaban group compared to placebo (4.2% versus 10.2%) was found. Of the patients included in the apixaban group, 4.8% had a brain tumor compared to 3.5% of the patients in the placebo group [49].

In the 2019 updated international clinical practice guidelines for the treatment and prophylaxis of VTE in cancer patients, the use of LMWH or unfractionated heparin commenced postoperatively for the prevention of VTE in patients with cancer and undergoing neurosurgery is recommended. For patients with brain tumors not undergoing neurosurgery, a primary pharmacological prophylaxis is not recommended [50]. This is consistent with the clinical guidance from the ISTH where no pharmacological prophylaxis is recommended for outpatients with brain tumors [51]. The European Society of Anesthesiology recommend for patients undergoing craniotomy with a high risk of VTE including malignancy, the initiation of mechanical thromboprophylaxis with intermittent pneumatic compression preoperatively in addition to LMWH or low dose unfractionated heparin postoperatively as soon as the bleeding risk is presumed to be decreased. The thromboprophylaxis is recommended to be continued until discharge [52].

### 4.2. Therapy

More-recently-updated guidelines for the treatment of VTE in cancer patients already include the data of the randomized controlled studies comparing LMWH and DOACs for their effectiveness and safety. Although there are slight differences, there is a general consensus. In patients with cancer- associated VTE, anticoagulation should be established with LMWH. In the absence of a high gastrointestinal and/or genitourinary bleeding risk, DOACs can also be used for the initial treatment of VTE in cancer patients. The anticoagulation should be given for 6 months. Thereafter an individual evaluation should be carried out for each patient including risk–benefit ratio, patient preference, and tumor activity [50,53].

Limited data regarding anticoagulation in patients with primary brain tumors leads to uncertainty with regard to which therapy is best for the effect–risk ratio in each patient. Current guidelines give support in the treatment of VTE in cancer patients but rarely offer recommendations especially for brain tumor patients (Table 2).

The American Society of Clinical Oncology states in its recently-published guidelines that in patients with primary brain tumors and VTE, anticoagulation should be offered, but uncertainty remains regarding choice of agent and patients most likely to benefit. Furthermore, limited safety data is available for the use of direct oral anticoagulants in patients with primary brain tumors [53].

In 2019, the international clinical practice guidelines for the treatment and prophylaxis of VTE in cancer patients recommended anticoagulation for established VTE in patients with primary brain tumors with LMWH or DOAC (grade 2B) [50].

## 5. Conclusions

Our understanding of the relationship between cancer, especially brain tumors, and VTE continues to improve as a result of insights from ongoing research. Brain tumor patients are at a high risk of VTE, a risk which is particularly high postoperatively but also remains high throughout the course of the disease. Recent guidelines recommend pharmacological thromboprophylaxis for the postoperative period. Nevertheless, brain tumor patients are not only at a high risk of VTE but also of bleeding events. Therapeutic anticoagulation is therefore challenging in this patient group. Particularly with regard to DOAC treatment, prospective data for these vulnerable patients is scarce. Risk stratification models can identify patients most at risk. Beyond the individual assessment of risk factors that are associated with the tumor, patient, or therapy biomarkers are needed to extend the risk stratification.

Highlighting patients with a high risk for VTE or a high bleeding risk, for example by biomarker, should be the subject of further studies.

## Figures and Tables

**Table 1:** Primary brain tumor or brain metastasis patients included in randomized clinical trials on efficacy and safety of direct oral anticoagulants (DOACs) versus low-molecular-weight heparin (LMWH) for treatment of cancer-associated venous thromboembolism (VTE).

Trial [Ref.]	DOAC Investigated	Brain Tumor or Brain Metastasis Patients Included, *n*	DOAC, *n*	Dalteparin, *n*
Hokusai VTE-cancer study [40]	Edoxaban	74	31	43
SELECT-D study [41]	Rivaroxaban	3	1	2
Adam-VTE study [42]	Apixaban	8	3	5
Caravaggio study [43]	Apixaban	excluded	x	x

Abbreviations: Ref. = Reference.

**Table 2:** Recommendations for prophylaxis and therapy of VTE for primary brain tumor patients according to current guidelines.

Guideline [Ref.]	Prophylaxis	Therapy
International Clinical Practice Guidelines [50]	Use of LMWH or unfractionated heparin commenced postoperatively for the prevention of VTE in patients with cancer and undergoing neurosurgery is recommended For patients with brain tumor not undergoing neurosurgery, a primary pharmacological prophylaxis is not recommended	Anticoagulation is recommended for established VTE with LMWH or DOAC
ISTH [51]	Pharmacological prophylaxis for outpatients with brain tumors is not recommended	-
ASCO [53]	−	Anticoagulation should be offered, but uncertainty remains regarding choice of agent Limited safety data is available for the use of direct oral anticoagulants
ESA [52]	Initiation of mechanical thromboprophylaxis with intermittent pneumatic compression preoperatively in addition to LMWH or low dose unfractionated heparin postoperatively as soon as the bleeding risk is presumed to be decreased Thromboprophylaxis is recommended to be continued until discharge	-

Abbreviations: Ref. = Reference; LMWH = low molecular weight heparin; VTE = venous thromboembolism; DOAC = direct oral anticoagulation; “−“ = no stated recommendation; ISTH = International Society on Thrombosis and Hemostasis; ASCO = American Society of Clinical Oncology; ESA = European Society of Anesthesiology.
